# Nordic walking training and nutritional supplementation in pre-frail older Indians: an open-labelled experimental pre-test and post-test pilot study to develop intervention model

**DOI:** 10.1186/s12877-018-0890-4

**Published:** 2018-09-14

**Authors:** Prasun Chatterjee, Prakash Kumar, Ramesh Kandel, Ruchika Madan, Meenakshi Tyagi, Deepa Anil Kumar, Maroof Ahmad Khan, Gaurav Desai, Preeti Chaudhary, Shyama Gupta, Kanika Grover, Aparajit Ballav Dey

**Affiliations:** 1 Department of Geriatric Medicine, All India Institute of Medical Sciences, New Delhi, India; 2 Division of Geriatric Medicine, Patan Academy of Health Sciences, Patan, Nepal; 3 Healthy Aging India, New Delhi, India; 4 Department of Biostatistics, All India Institute of Medical Sciences, New Delhi, India; 5 Corner Office Advisors, New Delhi, India; 6 University of Manitoba, Winnipeg, Canada

**Keywords:** Frailty, Adapted indoor Nordic walk, Individualized nutritional supplementation

## Abstract

**Background:**

Identifying and treating people in a pre-frail state may be an effective way to prevent or delay frailty and preserve their functional capacity. This study aimed to assess the efficacy of, and compliance with, a 12 week individualized nutritional supplementation (INS) and Nordic walking (NW) program in pre-frail older Indians. The primary measure is physical performance, as indicated by Fried’s Frailty scale. Other measures include: cognition, as indicated by the Hindi Mental Status Examination; mood, by the Geriatric Depression Scale; and nutritional status, by the Mini Nutritional Assessment.

**Methods:**

This is an open-labeled experimental pre-test and post-test study, which took place from October 2012 to December 2014. The study was approved by Institute Ethics committee (IEC/NP-350/2012/RP-26/2012) at the All India Institute of Medical Sciences (AIIMS), New Delhi. Participants were sixty-six pre-frail elderly, who were randomly allocated into three subgroups, namely: A (NW only), B (INS only), and C (NW and INS). One-way ANOVA was used to statistically assess differences in baseline characteristics for quantitative variables, with the Chi-Square/Fischer exact test utilized for qualitative variables. Paired t-tests were used to assess pre and post intervention difference within the group for quantitative variables, with McNemar’s Chi-Square test used for qualitative variables. Kruskal Wallis test was used to assess significant intervention effects among the groups. A *p*-value < 0.05 was considered as statistically significant.

**Results:**

There was significant effect of intervention in gait speed in group A (*p* = 0.001) and C (*p* = 0.002), but not in group B (*p* = 0.926). While there was no significant change in grip strength in Group A (*p* = 0.488) and B (*p* = 0.852), a statistically significant increase was observed in group C (*p* = 0.013). Mood significantly improved in group B (*p =* 0.025) and C *(p* = 0.021). No significant difference was noted in cognitive status across groups. Following the interventions, a total of 18.18% of pre-frail participants were classified as non-frail.

**Conclusions:**

Combining NW and INS provides a simple, pragmatic intervention with efficacy in the management of functionally vulnerable older adults, and allows their maintained independence. Future studies should replicate this readily applicable intervention in a larger cohort with a longer follow-up period.

**Trial registration:**

Clinical Trial Registry-India CTRI/2016/05/006937 [Registered on: 16/05/2016]; Trial was Registered Retrospectively.

## Background

Frailty syndrome is arguably the greatest challenge faced by an aging population. It also has obvious significance for clinicians, scientists and policy planners [1]. Frailty syndrome is a state of increased vulnerability due to poor resolution of homeostasis after a stressor event, thereby increasing the risk of adverse outcomes including falls, delirium and disability [2]. The paradigm of precision medicine may be readily applied to the concept of frailty, allowing its better management within main stream modern medicine [3]. The prevalence of frailty is variable across studies, due to differences in defining criteria and study samples. In community dwelling western population over the age of 65 years, a systematic review indicates an overall frailty prevalence of 10.7%, which increases with age, being evident in 15.7% in 80 to 84 year olds and 26% in those over 85 years of age [3].

‘Fitness’ and ‘frailty’ may be seen as forming the poles of a continuum [4], with the former indicating complete independence, and the latter indicating complete dependence. Age-associated progression along this continuum often results in stages from healthy, to a semi-robust, subclinical frailty, to a pre-frail and eventual frail state [5]. Frail populations have a substantially increased risk of falls, disability, and the need of institutional care as well as of mortality [6]. Systematic reviews indicate that the pre-frail state, an intermediate stage between non-frail and frail, to have a 41.6% prevalence in community samples, compared to the 10.7% prevalence of a frail state [7]. One study of an Indian tertiary care hospital shows a 21% frailty prevalence and a 20% pre-frailty prevalence, using Fried’s phenotypic criteria [8].

Pre-frail individuals have more than twice the risk of becoming frail compared to non-frail people [9]. Identifying and treating people in the pre-frail state is an opportunity to prevent or effectively delay frailty. Further, evidence suggests that older adults in a pre-frail, compared to a frail, state respond better to more intensive interventions [10].

Frailty has a multi-faceted pathogenesis and pathophysiology, with sarcopenia proposed to play an important role [8, 11]. Sarcopenia is a syndrome that is characterized by the progressive and generalized loss of skeletal muscle mass and strength, thereby increasing the risk of many adverse outcomes, including physical disability and poor quality of life, as well as and mortality [12]. Consequently, interventions focused on improving muscle strength should have utility. However, improving muscle strength can be challenging, given its dependence on multiple factors, such as nutritional status [13], and the functional capacity of the individual [14]. Numerous multi-factorial interventions consisting of nutritional supplementation and various forms of exercise have been studied in the frail elderly, with variable results [15–17]. Unfortunately, the compliance to regular exercise in frail older adults is low, due to intrinsic weakness, lack of inertia and the perceived risks of exercise [18]. Resistance training and/or diet seem the only effective interventions to treat or prevent sarcopenia in frail or pre-frail individuals [13].

**Fig. 1:**
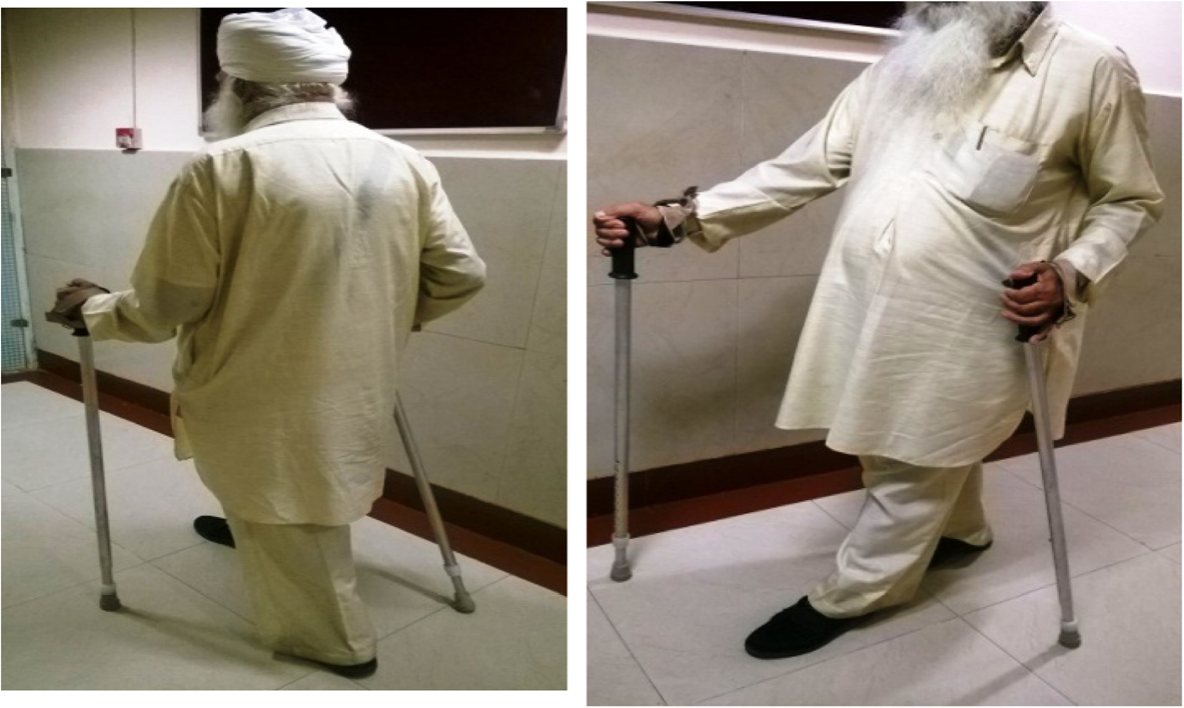
Adapted indoor Nordic Walk training

Walking is a rhythmic, dynamic, and aerobic activity with numerous benefits for the musculoskeletal system and almost no negative effects [19]. Nordic walking (NW) is a form of walking that uses muscles of the upper and lower body in a continuous and reciprocal movement [20] (Fig. 1). Due to the higher muscle mass involved, NW produces a higher cardio-respiratory workload, whilst improving endurance and flexibility as well as benefitting body balance functioning [21]. It is a whole-body aerobic or alternating aerobic/anaerobic discipline, rarely associated with physical injury and easily practiced by people of all ages, including older adults [15, 22–24]. A systematic review and meta-analysis by Bullo et al. summarizes the effectiveness of NW interventions on the physical fitness and quality of life in the elderly [25].

Inadequate dietary intake, especially of proteins and amino acids contributes to the development of sarcopenia and frailty [26–29], with a noticeable improvement in muscle strength occuring after protein and micro-nutrients supplementation [26]. Nutritional supplementation (protein and amino acid) in combination with physical training could further improve the functional status in frail populations. However, such work has focused on the combination of nutritional supplementation plus exercise for all study participants. It is important to clarify the relative contributions of exercise and diet. We hypothesized that, without pain and discomfort, an older person performing NW with individualized nutritional supplementation for 12 weeks would show a 20% greater improvement, versus either NW or INS alone. The current investigation is a pilot initiative study evaluating the efficacy of NW in functionally vulnerable older Indians, with or without INS, as well as in comparison to INS alone.

## Methods

This is an open-labeled experimental pre-post study from October 2012 to December 2014. The study was conducted with the approval of the Institute Ethics committee of All India Institute of Medical Sciences (AIIMS), New Delhi (Ref. No: IEC/NP-350/2012/RP-26/2012).

Participants: Individuals aged 60 years and above were recruited from the out-patient department of Geriatrics Medicine, AIIMS. Study inclusion criteria were: Pre-frail, as assessed by a Fried’s phenotypic scale [30] score of 1 or 2. Exlusion criteria: Any form of resistive training exercise or nutritional supplementation in the previous 6 months; having an acute illness, severe obstructive airway disease (Forced ExpiratoryVolume< 30%); severe systolic dysfunction (Ejection Fraction < 30% by Echocardiogram); severe depression (Geriatric Depression Scale > 11); severe, painful lower limb muscle condition; and severe cognitive impairment (Mini-Cog =0).

As per the out-patient department posting schedule of the first author (PC) and the principal investigator (ABD), three patients were randomly selected after obtaining consent to carry out frailty assessment. This was done three times a week for 18 months, giving a total of 648 patients. Of these 648 patients, 129 were found to be pre-frail as per Fried’s criteria. However, 63 patients declined to participate in the study, due to a variety of reasons, including transport, mobility issues and the lack of care giver support. Consequently, the 66 patients who gave informed written consent were recruited into the study.

**Fig. 2:**
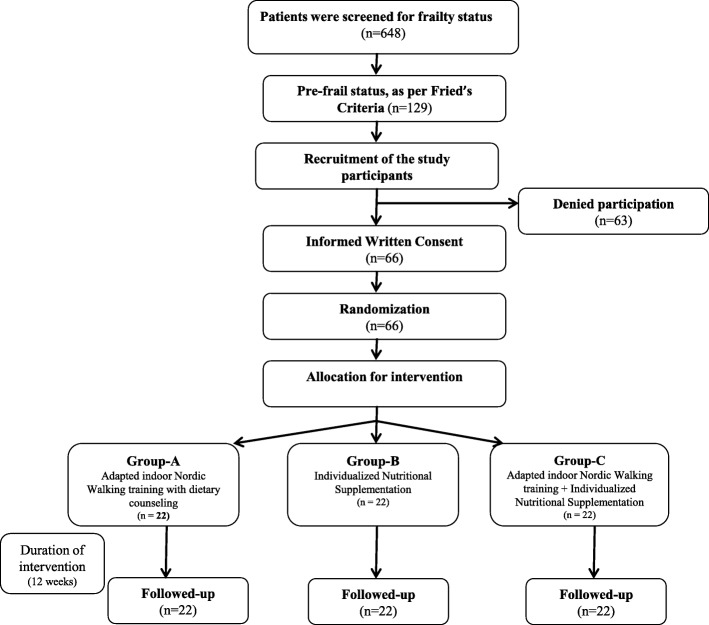
Flowchart showing the sequence of the study design

A standard randomization procedure for intervention allocation was generated using the n-query 2.0 software and allocation of the participants in three groups was done by sequentially numbered opaque sealed envelope. Sixty-six participants were therefore randomly allocated into three groups of twenty-two: Group A: NW; Group B: INS; and Group C: NW + INS (Fig. 2).

Demographic details including age, gender, education and income were obtained. A detailed assessment on nutritional status and physical performance was carried out by a qualified nutritionist (Masters degree in Food and Nutrition with two-years research experience in Geriatric Medicine) and an occupational therapist (Bachelors degree in Occupational Therapy with two years of clinical research experience in geriatric medicine), which provided baseline scores on these parameters.

Nutritional assessment was carried out by anthropometric measures, including weight, height, body mass index (BMI), and mid upper arm circumference (MUAC). A MUAC < 23.5 cm in males and < 22 cm in females was indicative of malnutrition. The nutritional status of the participants was assessed by Mini Nutritional Assessment (MNA®, Nestle Nutrition Institute) [31], an 18-point questionnaire with a maximum score of 24. Participants scoring < 17, 17–23.5 and 24–30 were labelled as ‘Malnourished’, ‘At risk of malnutrition’ and with ‘Normal nutritional status’ respectively.

To determine the concentration of serum albumin 5 ml blood was drawn from each study participant at baseline and after intervention. Serum was separated and albumin concentration was determined using standard protocols. Various functional assessments were performed. The assessment of the physical domain utilized the Modified Physical Performance Test (MPPT) and Berg Balance Scale (BBS). MPPT consists of nine components each with a minimum score of zero and maximum of 4 (total:36) [32]. Participants scoring between 0 and 16, 17–24, 25–31 and 32–36 are classified as severe, moderate, mild and non-frail, respectively. The BBS measure of balance consists of a 14-item scale designed to measure balance of the older adult in a clinical setting out of a total score of 56. Based on the scoring between 0 and 20, 21–40 and 41–56, participants were categorized into ‘low risk’, ‘medium risk’ and ‘high risk’ group respectively [33]. The assessment of daily living functioning utilized the ‘Barthel activity of daily living (BADL)’ and ‘Lawton and Brody instrumental activity of daily living (IADL).’ The BADL is an ordinal scale with 10 components, scored either 0 or 1, giving a total scale range of 0–20 [34]. The IADL consists of eight components, scored either 0 or 1, giving a total scale range of 0–8 [35]. Mood was assessed by 15-item short form of the geriatric depression scale (GDS), with a score over 5 denoting depression [36]. The cognitive domain was assessed by the ‘Hindi Mental Status Examination (HMSE)’ scale. The HMSE is a 23-item questionnaire with a maximum score of 30. Patients scoring less than 24 are regarded as cognitively impaired [37].

Frailty Assessment: Frailty was assessed by Fried’s phenotypical criteria [30], whereby one point is scored for each criterion met to specification. The components were: a) slow walking speed -walking 0.6 m/sec with or without a walking aid. b) Weakness (grip strength) was measured using hand dynamometer (in kg), taking the best of three attempts, with a grip strength of less than 6 kg in females and less than 15 kg in males as positive indicant of frailty. These cut-offs are also the 25th percentile of age matched control, which were taken as cut off value in previous published data from AIIMS [38]. c) An unintentional loss of weight greater than 5% of body weight or 10 lb. in the last one year was taken as positive indicant of frailty. d) Self Reported Exhaustion in the previous week, as indicated by response to being asked how often they felt that “everything they did was an effort” and “whether they could get going?”. Answer indicating this to be for3–4 days in the past week were taken as positive indicants of frailty. e) Low Physical Activity (over the past three months), with positive indicants of frailty being “not performing minimum weight-bearing physical activity” or “spending more than 6 hours per day sitting or lying other than sleep time.” Individuals with 3 or more of these characteristics were considered as frail, with those having 1 or 2 of these scale characteristics regarded as pre-frail.

### Adapted indoor Nordic walking training

The participants were trained for 12 weeks (36 training sessions each of 60 min duration), with a frequency of three times a week. Training sessions were individualized and provided by a qualified physiotherapist having Masters in Physiotherapy, with specialization in Orthopaedics, and using the NW technique according to the guidelines of the International Nordic Walking Federation (INWA) [39]. Each training session began with a short warm-up (5–10 min) which consisted of flexibility exercise for upper and lower extremities and spine. Training ended with stretching and breathing exercises (3–5 min). The pole length was adopted according to the participant’s physique, permitting a smooth arm motion and based on the INWA formula (0.7 X height cm). NW took place in a departmental corridor. Two walking poles were used to push against the ground with each stride, actively engaging the trunk and upper limbs during walking and maintaining natural gait, whilst the hands performed an open-close cycle in an alternating manner [15] (Fig. 1). Therapist walked side by side with the participant, providing encouragement throughout the walking sessions. During this training period, participants did not engage in any other physiotherapy and occupational therapy programs. Assessment of post-intervention dependent variables was done within 1 week of the final NW session.

Nutritional Intervention: Considering the high prevalence of malnutrition in India [40] the present study carried out assessment for both calorie and protein deficiency, with supplementation given as required. Calorie and protein requirement of each participant were assessed by a nutritionist. The participants from group A were counselled at baseline about their deficiency and were asked to improve their nutritional status by improving their diet. Participants belonging to group B and C were supplemented with the deficit quantity of nutrients, as per individual need, for 12 weeks. Carbohydrates were supplemented at the rate of ≈50% of total daily calorie requirement and protein was supplemented at the rate of 1.2 g/kg body weight. The objective of the supplementation was to provide an additional 100 Kcal with 8-10 g protein per day. Participants or their care-providers were called on weekly basis to provide nutritional supplementation powder. This gave the research team an indication of participant compliance towards nutrient supplementation, as well as any issues or complications that emerged. Participants were also instructed to return with the empty container after completion of the intervention.

#### Statistical analysis

Qualitative variables of participant characteristics were reported as numbers or percentages, whilst quantitative data were reported as the mean ± standard deviation (SD). Normality assumption among the groups was tested using the Kolmogorov-Smirnov Test. If normality was established, one-way ANOVA was carried out to see the association among the groups for quantitative variables and Chi-Square/Fischer Exact Test for qualitative variables. Paired t-tests were used to find the pre and post intervention differences within the group for quantitative data and McNemar’s Chi-Square Test for qualitative data. Kruskal Wallis Test was used to find the actual change effect among the groups. A *p*-value < 0.05 was considered as statistically significant.

## Results

The ages of participants (mean ± SD) in groups A, B, C were 75.77 ± 6.25, 76.88 ± 5.89 and 77.41 ± 3.78 years, respectively (*p =* 0.607). Table. 1 shows both quantitative and qualitative data at baseline. Similar baseline demographics were noted in each group and there were no statistically significant differences in age, gait speed, grip strength, BADL, IADL, serum albumin concentration and qualitative parameters such as GDS, HMSE, MNA, MPPT, BBS and frailty score.

**Table 1:** Baseline characteristics of the study participants

Variables	Group A (*n* = 22)	Group B (*n* = 22)	Group C (*n* = 22)	*p-* value
Sex				
Male	13	11	18	0.078
Female	9	11	4	
Age ± SD (years)	75.7 ± 6.24	76.68 ± 5.8	77.4 ± 3.78	0.607
Gait speed ± SD (metre/sec)	0.66 ± 0.1	0.67 ± 0.1	0.67 ± 0.1	0.970
Grip strength ± SD (Kg)	8.90 ± 2.1	9.09 ± 2.2	8.45 ± 2.32	0.620
BADL	3.95 ± 1.46	3.36 ± 0.90	3.77 ± 1.15	0.251
IADL	1.40 ± 0.50	1.36 ± 0.58	1.59 ± 1.00	0.555
Serum albumin (g/dl)	4.24 ± 0.48	3.97 ± 0.47	4.07 ± 0.55	0.207
GDS				
< 5	10	11	6	0.268
≥ 5	12	11	16	
HMSE				
< 24	10	9	9	0.940
≥ 24	12	13	13	
MNA				
Malnourished	9	9	12	0.518
At the risk	9	12	8	
Normal	4	1	2	
MPPT				
Severe	11	11	13	0.538
Moderate	6	9	7	
Mild	3	0	0	
Non-frail	2	2	2	
BBS				
Low - risk	5	5	5	1.000
Medium- risk	15	16	15	
High - risk	2	1	2	
Pre- frail status	22	22	22	0.676

Analysed by One-way ANOVA and Chi-square test

Participants’ gait speed (mean ± SD) in groups A, B and C were 0.66 ± 0.11, 0.67 ± 0.10 and 0.67 ± 0.11 respectively. There was significant effect of intervention on gait speed in group A (*p* = 0.001) and C (*p* = 0.002), but not in group B (*p* = 0.926). Grip strength (mean ± SD) in groups A, B and C were 8.91 ± 2.11 kg, 9.09 ± 2.20 kg and 8.45 ± 2.32 kg, respectively. No significant change was seen in grip strength in group A (*p =* 0.488) and B (*p =* 0.852), but a statistically significant increase was observed in group C (*p* = 0.013). Intervention resulted in statistically significant increase in BADL and IADL scores for all three groups. While a significant effect on MPPT score was observed only in group C (*p* = 0.020*),* whilst a significant effect of intervention on the balance measure, BBS, occurred in group A (*p* = 0.046) and C (*p =* 0.024), but not in group B (*p =* 0.270) (Table 2).

**Table 2:** Effect of intervention on different variables within groups

Variables	Categories	Group A			Group B			Group C		
		Pre	Post	*p*-value	Pre	Post	*p*-value	Pre	Post	*p*-value
Gait Speed		0.66 ± 0.11	0.99 ± 0.41	0.001	0.67 ± 0.11	0.66 ± 0.10	0.926	0.67 ± 0.10	1.00 ± 0.45	0.002
Grip Strength		8.9 ± 2.1	8.27 ± 3.9	0.488	9.09 ± 2.2	9.22 ± 2.5	0.852	8.45 ± 2.3	9.86 ± 2.8	0.013
BADL		3.95 ± 1.46	5.27 ± 1.20	<0.001	3.36 ± 0.90	4.81 ± 0.90	<0.001	3.77 ± 1.15	6.40 ± 0.95	<0.001
IADL		1.40 ± 0.50	2.59 ± 1.00	<0.001	1.36 ± 0.58	2.22 ± 0.81	<0.001	1.59 ± 1.00	3.31 ± 1.21	<0.001
Serum Albumin		4.24 ± 0.48	3.93 ± 0.48	< 0.001	3.97 ± 0.47	4.26 ± 0.5	<0.001	4.07 ± 0.5	4.40 ± 0.49	<0.001
MNA	Malnourished	9	12	0.050	9	1	0.005	12	0	0.003
	At the risk	9	9		12	20		8	17	
	Normal	4	1		1	1		2	5	
MPPT	Severe	11	6		11	8		13	2	
	Moderate	6	11	0.226	9	12	0.072	7	4	0.020
	Mild	3	4		0	2		0	12	
	Non-frail	2	1		2	0		2	4	
BBS	Low-risk	5	3		5	4		5	3	
	Medium-risk	15	10	0.046	16	14	0.270	15	10	0.024
	High-risk	2	9		1	4		2	9	
Frailty	Non-frail	0	2		0	4		0	6	
	Pre-frail	22	19	0.035	22	16	0.01	22	16	0.001
	Frail	0	1		0	2		0	0	
GDS	<5	10	9	0.739	11	16	0.025	6	14	0.021
	≥5	12	13		11	6		16	8	
HMSE	<24	12	13	0.564	13	12	0.564	13	10	0.083
	≥24	10	9		9	10		9	12	

Paired t-test and Mc Nemar’s Chi-Square test was performed to see the effect within the groups

There was statistically significant decrease in the serum albumin concentration in group A (*p* < 0.001), whilst groups B (*p* < 0.001) and C group (*p* < 0.001*)* showed significant increases. A statistically significant (*p* < 0.001) improvement in the nutritional status, as assessed by MNA, was also evident.

The effect of interventions on depression, as indicated by GDS score, showed significant improvement in groups B (*p* = 0.025) and C (*p =* 0.021). There was no significant impact on cognition across interventions.

Among the study groups, a significant difference was observed in gait speed between groups A and B (*p* = 0.003), B and C (*p =* 0.012) but the difference was not significant between A and C (*p* = 0.944). The difference in albumin concentration was significant among groups A and B (*p<0.001*) and groups A and C (*p<0.001*), but not significant among groups B and C (*p* = 0.603). A significant difference in BADL score was observed between group A and C (*p<0.001*), and groups B and C (*p<0.001*), but not between groups A and B (*p* = 0.677) (Table 3).

**Table 3:** Intervention effects across groups

Variables	Group A	GROUP B	GROUP C	*p*-value			
	Median (Min, Max)			A Vs B Vs C	A Vs B	A Vs C	B Vs C
Grip strength (Kg)	- 1.5 (−6, 7)	0.5 (−8, 5)	1 (−2, 7)	0.10	–	–	–
BADL	1.5 (0, 2)	1.5 (0, 3)	3 (1,3)	0.001	0.677	<0.001	<0.001
Serum albumin (g/dl)	−0.3 (− 1.1, 0.09)	0.25 (0, 0.7)	0.3 (−0.29, 0.8)	< 0.001	<0.001	<0.001	0.603
Gait speed (m/s)	0.33 (−0.33, 1.71)	0 (−0.2, 0.4)	0.21 (−0.3, 1.2)	0.007	0.003	0.944	0.012
IADL	0 (0, 2)	1 (0, 2)	3.72 (0, 4)	0.013	0.258	0.055	0.005

*p* < 0.016 was considered to be significant for multiple comparison (Kruskal-Wallis test followed by Wilcoxon Rankson test)

**Fig. 3:**
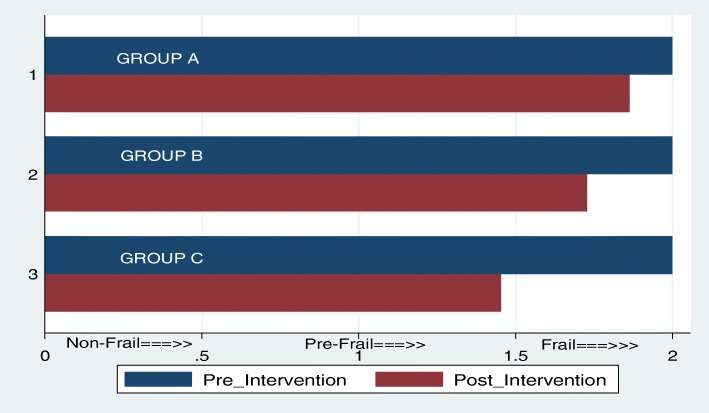
Frailty status on Pre and Post intervention

As shown in the Fig. 3, two participants (9.09%) in group A became non-frail, one became frail (4.55%) and the rest (86.36%) remained as prefrail. For group B these figure were 4 (18.18%), 2 (9.09%) and 16 (72.73%). In group C, 6 (27.27%) became non-frail whilst 16 (72.73%) remained pre-frail.

### Compliance with the protocol and adverse events

All of the randomized participants completed the trial. The values for median compliance within all three groups were also 100%. Diarrhea occurred in three participants receiving the nutritional supplement but they stopped the supplement for a week, and then restarted. No cardiovascular complications occurred during any testing or training sessions.

## Discussion

NW is a promising and practicable intervention for the maintenance of functionality in late life. In the present study, gait speed significantly improved with NW alone and in combination with INS. However, lone nutritional supplementation without NW did not show any impact on the gait speed of pre-frail adults. Our finding support previous report by Figueiredo et al., who observed an improvement in gait speed in older individuals following NW practice, versus an over-ground walking comparison group [20]. The combination of NW and INS also improved grip strength, although this effect was not evident for either alone. An earlier study reported a significant change in the grip strength of elderly women who practiced NW for the same duration as the present study [41]. This difference across studies may arise from variations in the levels of baseline malnutrition, 45.45% being malnourished and 43.94% at risk of malnourishment in the current study. Further, it might require a longer duration for lone nutritional supplementation to show any impact on muscle strength under variations in levels of malnourishment. The combined intervention seems to have an additive or synergistic effect in improving the strength of hand muscles.

All three groups showed improvements in functionality, indicating the utility of diet and NW on this parameter. Such data also highlights the importance of readily-modifiable nutritional deficiency in functional decline. Improvement of functional status is a primary goal for vulnerable older adults [42]. A study conducted by Persson et al. on the effects of combined nutritional treatments, in patients at risk of protein-energy malnutrition, also showed improved functionality as compared to controls [43].

**Fig. 4:**
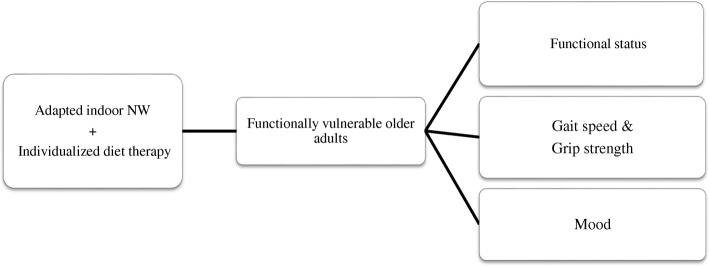
Changes in various domains after multimodal intervention

NW + INS and INS alone significantly improved participants’ mood. Many forms of exercise can improve mood, with some of the efficacy of exercise mediated via a lowering of the immune-inflammatory cascade that is evident in depression, as well as in pre-frail and frail older adults [44]. Previous work indicates the positive effect of NW on mood [44, 45]. However, the present study showed no mood improvement in the NW alone group. This again may arise from variations in study populations, with diet being more relevant to mood improvement in the current study. Previous studies have indicated that diet could regulate mood due to increased availability of key compounds, such as tryptophan, which is a necessary precursor or serotonin synthesis, thereby linking to the long-standing effects of serotonin in mood regulation [46]. In addition, small sample size and unmeasured factors/confounders may have contributed to the differences across studies. It is also of note, that due to the bidirectional association between depression and frailty [47], mood improvement may have a direct impact on the functionality of an individual and vice versa, possibly via immune-inflammatory regulation, including as arising from alterations in the microbiome and associated intestinal permeability. Many dietary factors can also act to regulate cognition, including more directly via brain neurotransmitter pathways, synaptic transmission, membrane fluidity and signal-transduction pathways [48], whilst diet and exercise may interact at the molecular level to influence cognitive abilities [48]. However, neither solitary nor multimodal interventions in the present study had any impact on cognitive status. This may have arisen due to the relatively short duration of the current pilot study, given that increased physical activity for at least 6 months may be necessary to improve cognition in older adults [49]. The current results also highlight the role of these simple interventions in the improvement of overall physical performance, as indicated by better frailty score in 18.18% of total study participants. However, intervention for a short duration might not show any significant impact on parameters such as weight loss, self reported physical exertions and physical performance (Fig. 4).

A recently published review of 17 studies of older adults (> 65 years) showed enhanced benefits of exercise training when combined with dietary supplementation [50]. This review also included studies of nutrition and exercise interventions aiming to impact on mass and/or muscle strength as indicants of improved physical performance [50].A study conducted by Rydwik et al. similarly showed a positive effect on lower-extremity muscle strength after a multi modal intervention [51]. The data of the present and previous studies are promising and of significant clinical importance, including as indicated by the comfort and high compliance with which older Indians practiced NW, coupled to no adverse events. Importantly, NW is regarded as a safe form of physical activity that helps in maintaining the body’s centre of gravity, thereby decreasing the risk of falls [52]. Further, NW can be performed by individuals who have relatively low levels of endurance and requires no added supervision. It is clear that this pilot study requires replication in a larger cohort and for a longer duration.

This is the first study to have focused on a simple, yet effective intervention model in pre-frail older adults from India. With proper health awareness among various society stakeholders, NW and INS can be incorporated as a healthy life style, thereby promoting active and healthy aging in late life.

### Limitation of the study

A major limitation of this study was that there was no non-treatment or control group. Further, this is a pilot study that clearly requires investigation in a larger cohort for a longer duration. Given the small sample size and the diversity of geriatric populations, the findings cannot be generalized.

## Conclusions

NW and INS were a simple pragmatic interventions to better optimize the benefits of exercise and nutrition on the functionally of vulnerable, pre-frail, older adults, allowing their greater independence. Although indications of some benefits are shown from NW and INS alone, there combination seems more effective in preventing the shift from a pre-frail to frail state. The current data need to be replicated and extended in a larger cohort with a longer follow-up period.

## Abbreviations

AIIMS: All India Institute of Medical Sciences
BADL: Barthel activity of daily living
BBS: Berg balance scale
BMI: Body mass index
GDS: Geriatric depression scale
HMSE: Hindi mental status examination
IADL: Instrumental activity of daily living
IEC: Institutional ethical committee
INS: Individualized nutritional supplementation
INWA: International Nordic walking federation
MNA: Mini nutritional assessment
MPPT: Modified physical performance test
MUAC: Mid upper arm circumference
NW: Nordic walking
SD: Standard deviation
